# Necrosis of the small intestine leading to a diagnosis of polyarteritis nodosa: a case report

**DOI:** 10.1186/s13256-019-2017-8

**Published:** 2019-03-09

**Authors:** Saori Yajima, Hiroshi Asano, Hiroyuki Fukano, Yasuhiro Ohara, Nozomi Shinozuka, Chih-Ping Li, Taketo Yamada

**Affiliations:** 1 Department of General Surgery, Saitama Medical University, 38 Morohongou, Moroyama, Iruma-gun, Saitama, 350-0495 Japan; 2 Department of Pathology, Saitama Medical University, 38 Morohongou, Moroyama, Iruma-gun, Saitama, 350-0495 Japan

**Keywords:** Polyarteritis nodosa, Intestinal necrosis, Necrotizing vasculitis

## Abstract

**Background:**

Polyarteritis nodosa is a disease that presents with necrotizing vasculitis in small and medium-sized arteries. It may occur in various organs, but approximately half of cases have gastrointestinal involvement. Prognosis is not favorable once organ dysfunction begins as evidenced by gastrointestinal symptoms; thus, treatment with steroids should be promptly initiated. We report the case of a patient who presented with necrosis of the small intestine, which was pathologically diagnosed as polyarteritis nodosa and treated successfully with steroids.

**Case presentation:**

An 18-year-old Japanese woman reported a sudden onset of abdominal pain and vomiting that led her to visit our emergency department, where she was evaluated by a physician. On physical examination, tenderness to palpation in the upper umbilical region was noted, and diagnostic imaging with computed tomography showed emphysema of the wall of her small intestine. She was diagnosed as having necrosis of the small intestine requiring urgent surgery. No strangulations were noted intraoperatively but approximately 20 cm of her small intestine was necrotized. The surrounding arteries were examined and no palpable pulse was observed; therefore, segmentectomy of the necrotized regions was performed. Pathological findings revealed active vasculitis with fibrinoid necrosis, as well as destruction, fibrogenesis, and luminal stenosis of the elastic lamina found in the muscular arteries. A diagnosis of polyarteritis nodosa was confirmed as the cause of the necrosis of her small intestine. No recurrence of polyarteritis nodosa symptoms was observed when she was administered 40 mg of prednisolone daily.

**Conclusion:**

In cases of idiopathic intestinal necrosis or perforation, systemic diseases such as polyarteritis nodosa should be considered in the differential diagnosis.

## Background

Polyarteritis nodosa (PAN) is a condition involving necrotizing vasculitis in small and medium-sized arteries [1]. It can often occur in various organs, with approximately half of patients having gastrointestinal involvement [2, 3]. Development of gastrointestinal lesions results from the formation of ulcers or erosions primarily due to ischemic changes with narrowing of the intravascular lumen. If perforation or necrosis is present, an accompanying acute abdominal condition should be considered because such cases require urgent surgery. In fact, some reports state that more than half of patients with PAN with associated abdominal symptoms require surgery for an acute abdominal condition [4]. Thus, patients may first present with an acute abdominal condition that leads to a postoperative diagnosis of PAN. PAN can be treated with steroids, but if organ dysfunction occurs, as evidenced by gastrointestinal symptoms, the prognosis is poor [5] and prompt treatment is desirable. For these reasons, patients with an acute abdominal condition, such as intestinal necrosis or perforation, should be treated with the consideration that PAN may be a possibility. The present patient underwent emergency surgery for suspected strangulated intestinal obstruction, which led to a definitive diagnosis of PAN after surgery, but it was also possible to suspect PAN based on the preoperative findings. We report a case who presented with necrosis of the small intestine, which was pathologically diagnosed as PAN and treated successfully with steroids.

## Case presentation

An 18-year-old Japanese woman experienced a sudden onset of abdominal pain and vomiting, and was therefore transported to the out-patient emergency ward at our institution. She was a student with no employment history. Her height was 156 cm and weight was 55 kg. Her consciousness was normal and there were no neurological abnormalities. She had been receiving antihistamines for atopic dermatitis but had no other conditions, such as neuropathy or hematuria, which would lead us to suspect neuritis or vasculitis. She had no history of tobacco smoking or alcohol consumption. Moreover, there was nothing of note in her family history.

**Fig. 1:**
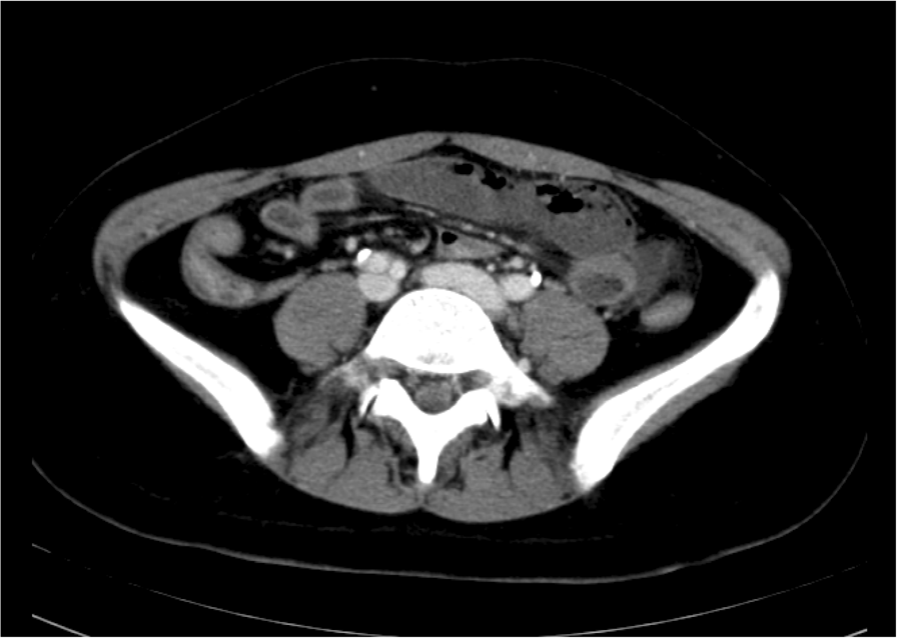
Findings on abdominal contrast-enhanced computed tomography. Poor contrast enhancement of the small intestine is noted

**Fig. 2:**
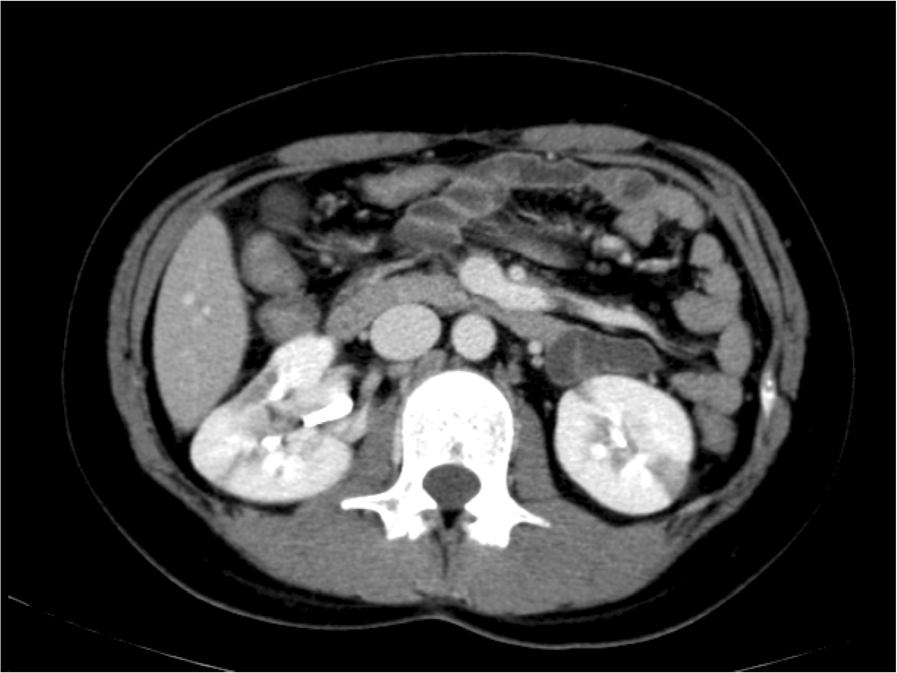
Findings on abdominal contrast computed tomography. Regions with poor contrast enhancement are noted in both kidneys

On physical examination, palpation revealed a flat abdomen with tenderness in the upper umbilical region, and no signs of peritoneal irritation. Her temperature was 36.3 °C, blood pressure was 159/123 mmHg, pulse was 85 beats/minute, and oxygen saturation was 99% on room air. Her blood laboratory findings revealed a white blood cell count of 8600/mm^3^ and a C-reactive protein level of 0.12 mg/dL, which was not suggestive of an inflammatory process. However, an increased D-dimer level of 4.36 μg/mL was noted. Her renal and liver functions were normal (blood urea nitrogen 9 mg/dL, creatinine 0.4 mg/dL, aspartate aminotransferase 31 U/L, and alanine aminotransferase 21 U/L). An abdominal contrast-enhanced computed tomography (CT) scan showed emphysema of the wall of her small intestine with poor contrast enhancement that corresponded to the area of tenderness (Fig. 1). However, the contrast CT revealed heterogeneous contrast enhancement, leading to a suspicion of renal infarction (Fig. 2). Necrosis of the small intestine was suspected based on the imaging findings, and urgent surgery was performed.

**Fig. 3:**
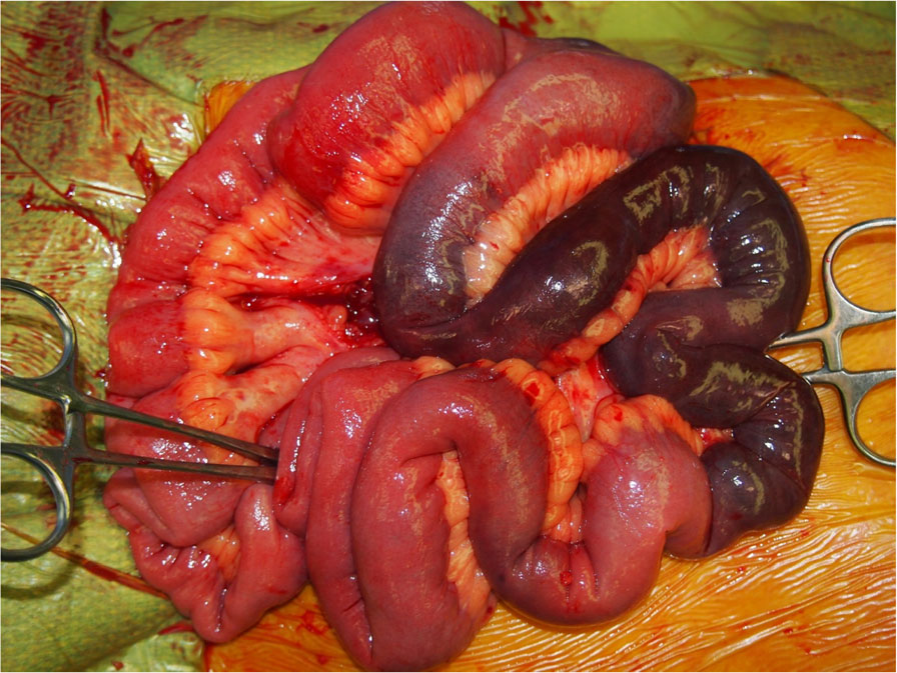
Intraoperative findings. The small intestine with approximately 20 cm of necrotized tissue

Laparotomy revealed an area of necrosis 20 cm in length, which was approximately 100 cm away from the Treitz ligament of the jejunum. There were no findings of strangulation, which precluded the identification of the cause of the necrosis (Fig. 3). Since there was no palpable pulse over the arteries surrounding the necrotized intestine, the necrotized regions were resected and the small intestine reconstructed to maintain a palpable pulse.

**Fig. 4:**
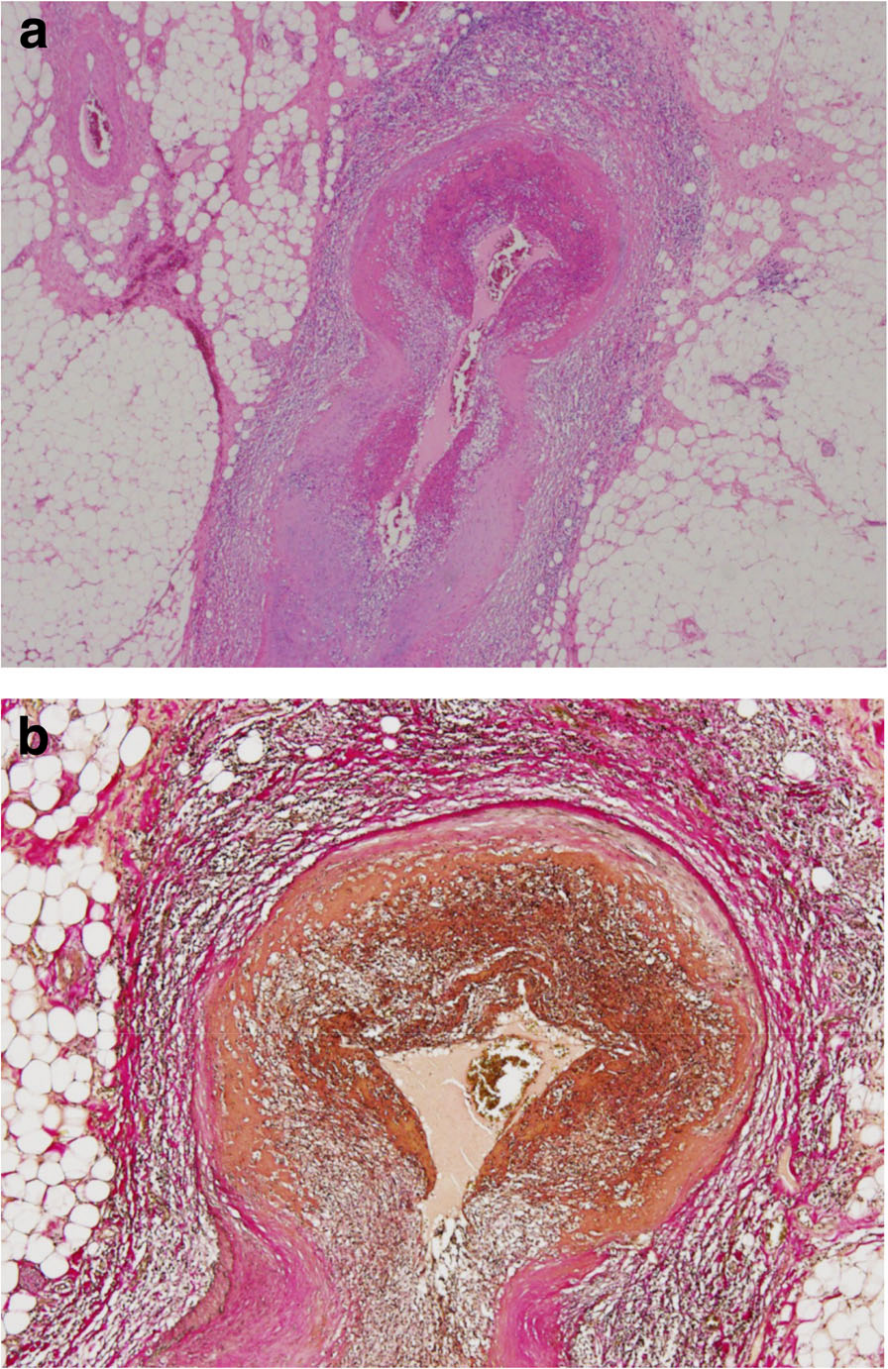
Histopathological findings. **a** Active vasculitis associated with fibrinoid necrosis was seen in the mesenteric artery (× 20). **b** Elastica van Gieson staining showed destruction, fibrogenesis, and luminal stenosis of the elastic lamina (× 40)

After surgery, a thrombotic lesion was suspected and anticoagulant therapy with heparin sodium at 20,000 U/day was started. However, pathological findings showed active vasculitis associated with fibrinoid necrosis, as well as destruction, fibrogenesis, and luminal stenosis of the elastic lamina in the muscular arteries. Our patient was therefore diagnosed as having PAN-induced necrosis of the small intestine (Fig. 4). Other postoperative investigations included a blood test, which showed that she was negative for proteinase 3-antineutrophil cytoplasmic antibody (ANCA) and hepatitis B surface antigen, but weakly positive for myeloperoxidase (MPO)-ANCA (5.3 U/mL; normal range, 0.0–3.4 U/mL). A postoperative angiograph of her renal arteries demonstrated multiple aneurysms in the renal arterioles. Postoperative urine analysis was positive for proteinuria. Anticoagulant therapy was discontinued, and she was administered 40 mg of prednisolone daily starting on postoperative day 11. Since she continued to improve, the dose of prednisolone was reduced. It has been a year since the surgery, and she is receiving prednisolone at 5 mg a day without recurrence.

## Discussion

In this case, emergency surgery was performed based on a diagnosis of necrosis of the small intestine, caused by an internal hernia or strangulated intestinal obstruction, and PAN was diagnosed pathologically after the operation. Although PAN is often accompanied by gastrointestinal symptoms, few cases develop due to intestinal necrosis. Similar to this case, emergency surgery based on a diagnosis of non-occlusive mesenteric ischemia [6] or mesenteric artery thrombosis [7] has been reported. Previous studies also indicate that diagnosing PAN preoperatively in the presence of an acute abdomen is difficult and that it is commonly diagnosed after postoperative pathological examination. While preoperative diagnosis was not possible in this case, in retrospect, PAN may have been suspected preoperatively based on the presence of renal infarction. As early steroid therapy improves the prognosis of PAN, early consideration should be given to systemic diseases when diagnosing cases of intestinal necrosis.

PAN is a systemic vasculitis disorder that presents with necrotizing vasculitis in small and medium-sized arteries, without arteriolitis or capillaritis. Patients with PAN are also classically MPO-ANCA-negative. However, MPO-ANCA-positive cases involve vasculitis of the arterioles and capillaries, and are therefore diagnosed as microscopic polyangiitis. However, there have been reports of MPO-ANCA-positive PAN cases [8, 9]. These cases were diagnosed as PAN because the pathological findings showed necrotizing vasculitis in small and medium-sized arteries despite being MPO-ANCA-positive. In fact, in the present case, angiographic and pathological findings indicated lesions in the arterioles; thus, our patient was diagnosed as having PAN despite MPO-ANCA positivity.

Patients with PAN show a wide variety of symptoms, such as fever, weight loss, malaise, multiple arthralgia, myalgia, and muscle weakness. PAN can be diagnosed based on these clinical findings, as well as by the presence of necrotizing arteritis in the muscular arteries on histological investigation. It often occurs in various organs, with the kidneys more likely to be affected. This results in the onset of renal failure due to vasculitis-induced renal infarction. The digestive organs are also common sites of PAN, with half of patients with the disease having gastrointestinal lesions. Lesions of the small intestine are frequently present, but lesions can also occur in the large intestine, liver, pancreas, and gallbladder [8].

Gastrointestinal lesions are characterized by narrowing of the intravascular lumen due to vasculitis, with the formation of ulcers or erosions observed on gross pathology. Patients with PAN commonly suffer from non-specific symptoms, such as abdominal pain, nausea, or diarrhea, with some reports showing that 14–25% of patients with PAN-related gastrointestinal lesions initially experience gastrointestinal symptoms [9, 10]. As the lesions progress, bleeding or perforation can lead to ischemia and, eventually, necrosis. Levine *et al.* [4] investigated 24 cases of PAN with gastrointestinal symptoms and found that 54% had acute abdominal conditions requiring surgery, including eight that had progressed to necrosis or perforation and four with bleeding caused by a ruptured aneurysm.

According to Bourgarit *et al.* [5], there are five risk factors that impact the prognosis of PAN, including uric protein > 1 g/day, serum creatinine levels > 1.58 mg, prior gastrointestinal surgery, cardiomyopathy, and a central nervous system disorder. In Bourgarit *et al*.’s study, the 5-year survival rate was 88% in patients with zero risk factors, which decreased to 55% in those with three or more risk factors. Pagnoux *et al*. [3] reported on 62 cases with necrotizing vasculitis, including 38 cases of PAN with a 5-year survival rate of 76%, among which 50% of mortalities were caused by gastrointestinal lesions. Other reports have revealed a 5-year survival rate of 67% in cases without gastrointestinal lesions, and 55% in those complicated with gastrointestinal lesions [11]. As mentioned above, the prognosis of PAN is not encouraging; thus, careful follow-up including recurrence prevention is required, particularly for cases complicated by gastrointestinal lesions.

Regarding treatment, the steroids prednisone or prednisolone are used, with doses usually starting at 1 mg/kg per day. The dose can then be reduced as needed if the patient’s condition improves. However, in cases where disease recurrence precludes steroid reduction or where organ dysfunction has occurred, the combined use of immunosuppressive agents may be considered. In addition, in cases of life-threatening conditions or rapid disease progression, steroid pulse therapy may be applied [2]. In this case, our patient was started on 40 mg of prednisolone on postoperative day 11 and she has had no recurrence of gastrointestinal lesions in the remaining intestinal regions. Since her condition was stable, no immunosuppressive agents were administered.

## Conclusion

Since PAN is associated with a high incidence of gastrointestinal lesions, an acute abdominal condition may occur as the initial presentation. A pathological assessment is required for the diagnosis of PAN. In cases of intestinal necrosis or perforation where intraoperative findings cannot clarify the cause, sample collection is necessary to definitively diagnose a systemic disease such as PAN.

## Abbreviations

ANCA: Antineutrophil cytoplasmic antibody
CT: Computed tomography
MPO-ANCA: Myeloperoxidase-antineutrophil cytoplasmic antibody
PAN: Polyarteritis nodosa
